# Hypersensitivity to non-steroidal anti-inflammatory drugs (NSAIDs): classification of a Danish patient cohort according to EAACI/ENDA guidelines

**DOI:** 10.1186/s13601-015-0052-0

**Published:** 2015-03-03

**Authors:** Christoffer V Nissen, Carsten Bindslev-Jensen, Charlotte G Mortz

**Affiliations:** Department of Dermatology and Allergy Centre, Odense Research Center for Anaphylaxis (ORCA), Odense University Hospital, University of Southern Denmark, Sdr. Boulevard 29, DK-5000 Odense C, Denmark

**Keywords:** Non-steroidal anti-inflammatory drugs, Classification, Hypersensitivity, Drug allergy, Provocation test

## Abstract

**Background:**

Non-steroidal anti-inflammatory drugs (NSAIDs) are reported to be the second most common cause of drug hypersensitivity. In 2011, experts from the EAACI/ENDA group and GA^2^LEN proposed a new classification system for NSAID hypersensitivity. The aim of this study was to classify a patient cohort with a history of NSAID hypersensitivity according to this system.

**Methods:**

Patients with a clinical history of NSAID hypersensitivity referred to the Allergy Centre, Odense University Hospital between 2002 and 2011 and evaluated with oral provocation tests (OPTs) were included in the study. Medical records were retrospectively investigated with respect to the culprit NSAID(s), underlying diseases and symptoms at the primary reaction and during oral provocation tests (OPTs). Data was supplemented with a questionnaire. Classification according to EAACI guideline was based on these findings.

**Results:**

In total 149 patients were included. Of those, 39 patients (26.2%) had a positive OPT. Twenty-nine patients were classified as cross-reactive responders and 9 patients as single NSAID responders after positive OPTs with the culprit NSAID, but not to acetylsalicylic acid. All single NSAID responders reacted to non-pyrazolone drugs. Only one patient could not be classified according to the EAACI/ENDA system. An overlap between respiratory and cutaneous symptoms was found in 15/39 (38%) of patients.

**Conclusions:**

All but one of our patients could be classified according to the EAACI classification system. Overlaps between different classes may occur much more commonly than expected.

## Background

Non-steroidal anti-inflammatory drugs (NSAIDs) are among the most frequently prescribed classes of drugs and hypersensitivity to NSAIDs is reported to be the second most common cause of drug hypersensitivity [1]. Aspirin hypersensitivity alone is estimated to affect 0.3%-2.5% of the general population [2,3]. However, the prevalence of NSAID hypersensitivity among patients with underlying diseases such as asthma and chronic urticaria is much higher, affecting up to 25% and 30% respectively [4,5].

Several subtypes of NSAID hypersensitivity have been described depending on symptoms (respiratory, cutaneous, anaphylaxis), timing (immediate, delayed), underlying chronic diseases (asthma, chronic urticaria) or the possible mechanism of the reaction (allergic, non-allergic) [6-8]. Subsequently, attempts have been made to classify NSAID hypersensitivity reactions based on clinical history and results of drug provocation tests [9,10]. However, until recently no uniform definition or classification of NSAID hypersensitivity had been proposed. This changed in 2011 where experts from the EAACI/ENDA group and GA^2^LEN proposed a new classification system for NSAID hypersensitivity launching guidelines on diagnostic work-up and management [11]. In 2013, the authors proposed further unification of this classification and a revised nomenclature as well as graded recommendations [12]. The new classification system is based on knowledge about type of reaction, clinical manifestation, timing of the reaction, underlying diseases, cross-reactivity and putative mechanisms.

A detailed clinical history is essential when planning the diagnostic work-up. However, to confirm the diagnosis NSAID hypersensitivity an oral challenge test is needed when taking test indications, contraindications and security aspects into account. In vivo and in vitro tests are not currently recommended as a routine practise except for the non-cross reactive IgE mediated reactions [12].

During the last 10 years we have evaluated consecutive patients referred with suspicion of NSAID hypersensitivity with standardized challenge tests. We used the results of the challenge tests and our knowledge about the patients’ clinical history to classify the patients with NSAID hypersensitivity according to the EAACI/ENDA guideline. This is the first study from the northern part of Europe classifying NSAID hypersensitivity in a standardized way based on history, clinical examination, oral challenge and questionnaire data.

## Methods

### Patient selection

Patients with a clinical history of NSAID hypersensitivity referred to the Allergy Centre, Odense University Hospital, Denmark, between 2002 and 2011 were retrospectively identified from our database. Patients where diagnostic work-up suggested acute cutaneous, respiratory and/or anaphylactic reactions in relation to NSAID intake were referred to an oral provocation test (OPT) and included in the study. None of the included patients had a history of severe cutaneous adverse drug reactions (SCARs). Patients with an isolated reaction to paracetamol were not included.

### Medical records and questionnaire

Medical records of the included patients were investigated with regards to the culprit NSAID(s). Symptoms at the primary reaction and during OPTs were analysed. Anaphylaxis was defined as a rapidly evolving reaction involving multiple organs in accordance to Simons [13] and the severity was graded according to Sampson [14].

Data on underlying chronic urticaria, angioedema, asthma, nasal polyposis and chronic rhinosinusitis (CRS) were collected from the medical records. Furthermore, a modified version of the questionnaire used in the GA^2^LEN survey was sent by post to the patients [15]. This questionnaire covers a wide range of questions concerning asthma, chronic rhinosinusitis and allergic rhinitis. In addition to the existing questions we added the following questions: “Have your doctor ever told you that you have enlarged nasal polyps?” and “Have you ever had nasal polyps removed?” Questionnaire surveys are exempted from notification of the Danish Ethical Committees Act §8 paragraph 3.

In concordance with the GA^2^LEN survey, asthma was defined as reporting having ever had asthma AND at least one of the following symptoms in the last 12 months (i) wheeze or whistling in the chest (ii) waking up with chest tightness (iii) waking up with shortness of breath and (iv) waking up with an attack of cough.

Chronic rhinosinusitis was defined following the EP3OS criteria: Presence of at least two of the following symptoms for at least 12 weeks in the past year: (i) nasal blockage, (ii) nasal discharge, (iii) facial pain or pressure or (iv) reduction in sense of smell with at least one of the symptoms being nasal blockage or nasal discharge.

### Oral provocation test (OPT)

Included patients performed a primary OPT with acetylsalicylic acid (ASA). If the OPT was positive no further challenges were performed. If the OPT was negative the patient was offered an OPT with the culprit NSAID. All OPTs were performed as open challenges under anaphylaxis surveillance after informed consent of the patient. During provocation with ASA and naproxen the following dosages were given orally with 30-minute intervals (5 mg, 25 mg, 125 and 500 mg) adding up to a cumulative dosage of 655 mg. Ibuprofen and diclofenac were also administered with 30-minute intervals but in different dosages, ibuprofen (5 mg, 25 mg, 125 mg and 400 mg) and diclofenac (0.5 mg, 5 mg and 50 mg). After every dose the patient was assessed and the OPT was stopped when an objective clinical reaction occurred – i.e. urticaria, angioedema, asthma, rhinoconjunctivitis or anaphylaxis. When symptoms were suggestive of hypersensitivity but without objective findings, the previous dosage was either repeated or the following dosage lowered. This accounts for the variation in threshold dosages (Table 1). Patients were observed 2 hours after the last dosage was given. In patients suffering from chronic urticaria the disease had to be without spontaneous attacks requiring antihistamine for at least one month before challenge. In all other patients, antihistamines and other drugs likely to affect the outcome were discontinued minimum three days before the OPT. All patients with a positive OPT were subsequently classified according to EAACI/ENDA classification system for NSAID hypersensitivity reactions [11] and to a recent more simple classification system [16].

**Table 1:** **Threshold dosages for positive OPTs with acetylicsalicylic acid**

**Threshold dosage (mg)**	750*	667.5*	**655**	500*	185*	**155**	80*	55*	**30**	**5**
**Patients n = 30**	1	1	18	1	1	3	1	1	2	1

Following dosages were given orally with 30-minute intervals (5 mg, 25 mg, 125 and 500 mg) adding up to a cumulative dosage of 655 mg.

Mean threshold dosage: 485.3 mg.

*Dosage in regime either repeated or lowered during OPT due to subjective complaints from the patient. See Methods.

OPT = oral provocation test.

### Statistics

The questionnaire responses and clinical data were entered into the database by the first author. Statistical analysis was performed with STATA/SE 11.0 (Stata Corporation, TX, USA).

## Results

A total of 149 patients, 105 women (70.5%) and 44 men (29.5%) with suspected NSAID hypersensitivity, were referred to OPT. The mean age was 44.5 years (range 12–80 years).

Patients’ clinical history revealed that ASA and ibuprofen were the two most common causes of reported NSAID hypersensitivity reactions comprising 40% and 32% respectively (Table 2). Urticaria/angioedema was the most frequently reported symptom (51.0%), whereas reactions exclusively involving the airways i.e. asthma/rhino-conjunctivitis were rare (7.4%). Not surprisingly, patients often reported symptoms from different organ systems simultaneously with urticaria/angioedema and asthma/rhinoconjunctivitis being the predominant symptom complex (23.5%). Anaphylaxis defined as grade 3–5 reactions according to severity score proposed by Sampson was reported by 11.4% of the patients (Table 2).

**Table 2:** **Symptoms and reported eliciting drug(s) based on case history of patients suspected of NSAID hypersensitivity**

			**Symptoms**						
		**Patients n = 149 (%)**	**Urticaria/ angioedema n = 76 (51.0%)**	**Asthma/rhino-conjunctivitis n = 11 (7.4%)**	**Urticaria/ angioedema + asthma/ rhino- conjunctivitis n = 35 (23.5%)**	**Anaphylaxis n = 17 (11.4%)**	**Non-urticaria rash n = 6 (4.0%)**	**Non-urticaria rash + asthma/rhino-conjunctivitis n = 3 (2.0%)**	**Other Nasal polyposis n = 1 (0.7%)**
**Suspected drug**	**Acetylsalicylic acid**	**59 (40)**	33	5	12	5	3	-	1
	**Ibuprofen**	**48 (32)**	26	2	8	8	2	2	-
	**Diclofenac**	**20 (13)**	6	1	7	4	1	1	-
	**Mulitple NSAIDs**	**18 (12)**	9	3	6	-	-	-	-
	**Naproxen**	**2 (1,5)**	1	-	1	-	-	-	-
	**Miscellaneous NSAID**	**2 (1,5)**	1	-	1	-	-	-	-

**Figure 1:**
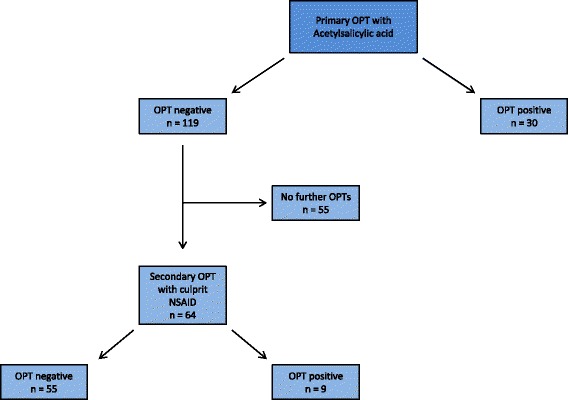
**Approach to OPTs in 149 patients suspected of NSAID hypersensitivity.** All patients performed a primary OPT with acetylsalicylic acid. If the primary OPT was negative, and ASA not was the culprit drug, a secondary OPT was performed with the culprit NSAID according to case history. See Figure 2 for details about the secondary OPT. OPTs = oral provocation tests.

**Figure 2:**
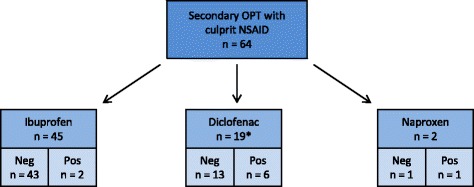
**Results of secondary oral provocation test (OPT) with culprit NSAID.** Sixty-six OPTs were performed in 64 patients with a negative challenge for ASA. Nine patients had a positive secondary OPT. *Two patients had a history of multiple reactions after intake of several NSAIDs and underwent provocation tests with ibuprofen and diclofenac, both of which were negative. OPTs = oral provocation tests.

All patients had a primary titrated oral provocation with ASA resulting in 30 positive reactions (Figure 1). If the primary OPT was negative, and ASA not was the culprit drug, a secondary provocation test was performed. Hence 55 patients did not undergo further provocation tests whereas 64 patients were challenged with their culprit NSAID according to case history (ibuprofen, diclofenac or naproxen). These provocation tests yielded 9 positive and 55 negative reactions. Two patients had a history of multiple reactions after intake of several NSAIDs and underwent provocation tests with both ibuprofen and diclofenac, both of which were negative (Figure 2).

In total, OPTs were positive in 39/149 tested patients (26.2%). The distribution of positive reactions was as follows: thirty ASA, six diclofenac, two ibuprofen, and one naproxen. Table 3 presents the relationship between case history and positive OPTs with respect to type of reaction. The most common symptom during OPT was urticaria/angioedema either alone (17 patients, 43.6%) or in combination with respiratory symptoms (15 patients, 38.5%). Respiratory symptoms as a solitary sign were seen in 5 patients (12.8%). Anaphylaxis was observed in 2 patients (5.1%) both of whom during testing with diclofenac. A high degree of concordance was found when comparing the symptoms of the primary reaction with the symptoms of the OPT with 67% of cases having full concordance and 23% having partial concordance (Table 3).

**Table 3:** **Relationship between case history and oral provocation test with respect to type of reaction**

		**Case history**			
		**Urticaria angioedema**	**Asthma/rhino-conjunctivitis**	**Urticaria/angioedema+asthma/rhino-conjunctivitis**	**Anaphylaxis**
Oral provocation test (OPT)	Urticaria/angioedema n = 17	13^a^		3^b^	1
	Asthma/rhinoconjunctivitis n = 5		5^a^		
	Urticaria/angioedema + Asthma/ rhino-conjunctivitis n = 15	6^b^		8^a^	1
	Anaphylaxis n = 2			2	

^a^Indicate concordance between case history and oral provocation test. ^b^Indicate partial concordance between case history and OPT.

The threshold dosage for eliciting a positive reaction during OPT for ASA is presented in Table 1. Mean threshold among the 30 positive ASA was 485.3 mg. High cumulative dosages were also needed to elicit positive reactions in OPTs with naproxen (655 mg), ibuprofen (570 mg) and diclofenac (36.8 mg).

Our questionnaire was sent to 147/149 patients; 2 patients had died between their diagnostic work-up and data collection. The response rate was 81% (119 patients). Of the 39 patients with a positive OPT, 32 replied.

Based on the positive OPTs and our knowledge of the patients’ underlying diseases collected from the questionnaire and medical records we were able to classify all but one patient according to the EAACI/ENDA guidelines (Table 4) and to the classification system published by Caimmi et al. (Table 5). Using the EAACI/ENDA guidelines 4 patients in the NERD group also had cutaneous symptoms and in the NECD and NIUA group 11 patients also had respiratory symptoms.

**Table 4:** **Characteristics of 39 patients with positive OPT according to the EAACI/ENDA classification** [12]

**Type of reaction**	**Clinical manifestation**	**Timing of reaction**	**Underlying disease**	**Cross-reactivity**	**Putative mechanism**	Patients n = 38*
NSAIDs-exacerbated respitoratory disease (NERD)	Bronchial obstruction, dyspnea and/or nasal congestion/rhinorrhea	Acute (usually immediate to several hours after exposure)	Asthma/rhinosinuitis/nasal polyps	Cross-reactive	Non-allergic COX-1 Inhibition	9
NSAIDs-exacerbated cutaneous disease (NECD)	Wheals and/or angioedema		Chronic urticaria	Cross-reactive	Non-allergic COX-1 Inhibition	14
NSAIDs-induced urticaria/angioedema (NIUA)	Wheals and/or angioedema		No underlying chronic diseases	Cross-reactive	Non-allergic Unknown, probably COX-1 inhibition	6
Single NSAID-induced urticaria/angioedema and anaphylaxis (SNIUAA)	Wheals/angioedema/anaphylaxis		No underlying chronic diseases	Single drug induced	Allergic IgE-mediated	9
Single-NSAID-induced delayed reactions (SNIDR)	Various symptoms and organs involved (e.g., fixed drug eruption, SJS/TEN, nephritis)	Delayed onset (usually more than 24 h after exposure)	No underlying chronic diseases	Single drug or multiple drug induced	Allergic T-cell mediated	0

*1 patient could not be classified.

**Table 5:** **Characteristics of 39 patients with positive OPTs according to the Caimmi classification** [16]

**Time of reaction**	**Clinical manifestation**	**Type of reaction**	**Underlying disease**	**Putative mechanism**	Patients n = 38*
Acute (immediate to several hours after exposure)	Rhinitis-asthma and/or urticaria-angioedema	Cross-reactive	Asthma/rhinosinuitis and/or chronic urticaria	Inhibition of COX-1	23
	Urticaria/angioedema and/or rhinoconjunctivitis	Multiple NSAIDs induced	No underlying chronic disease or atopy or rhinoconjunctivitis	Unknown, presumably related to COX-1 inhibition	6
	Urticaria/angioedema/anaphylaxis	Single drug induced	Atopy or food allergy or drug allergy	IgE-mediated	9
Delayed (more than 6 h after exposure)	Fixed drug eruptions, severe bullous skin reaction, maculopapular drug eruptions, pneumonitis, aseptic meningitis, nephritis, contact and photo-contact dermatitis	Single drug or multiple drug induced	Usually no	T-cell mediated (type IV) Cytotoxic T cells NK cells Other	0

*1 patient could not be classified.

## Discussion

### Value of the clinical history

Diagnosing NSAID hypersensitivity is challenging. As proposed in the revised EAACI/ENDA guideline [12] a detailed clinical history with regards to previous hypersensitivity reaction(s) and underlying diseases is essential when planning the diagnostic work-up.

The value of the clinical history to establish the diagnosis of NSAID hypersensitivity remains, however, controversial. In a recent study by Blanca-Lopez et al. [17], oral provocation tests confirmed the diagnosis of multiple NSAID-triggered urticaria/angioedema in 92% of cases when more than two NSAIDs were involved in the clinical history. Dursun et al. [18] also showed good concordance between patient history and results of OPTs in 243 patients with varying severity of asthma. A total of 86% of the patients with a clinical history of NSAIDs-exacerbated respiratory disease (NERD) had a positive OPT. The impressive results in both studies were reached analysing selected patient populations. The patient population in our study was more diverse and only 18 patients (12%) had a history of multiple NSAIDs eliciting hypersensitivity reactions (Table 2). This might explain why our results differ considerably from those above. Only 39/149 patients (26.2%) had a positive OPT despite having a relevant clinical history. Previous studies with unselected patients by Schubert et al. [19] and Viola et al. [20] showed similar concordance rates between patient history and positive provocation tests, 13.8% and 22.2% respectively. Our findings confirm that OPT is the most reliable method to establish the diagnosis of NSAID hypersensitivity in an unselected patient population [11,21].

Recent work by our research group revealed a high correlation between case histories and reactions elicited during OPTs in patients with penicillin allergy [22]. In the present study, the confirmed NSAID hypersensitive patients also showed a high degree of correlation between case history and reactions upon OPTs (Table 3). Although the case history is not reliable when diagnosing NSAID hypersensitivity, the case history might predict the pattern of response in actual NSAID responders.

All patients in our cohort underwent OPT regardless of the severity of their clinical history. This included 17 patients with a clinical history of anaphylaxis (Table 2). Subsequently, only two patients had a positive reaction upon OPT none of which were anaphylaxis (Table 3). This suggests that the severity of the clinical history is not a reliable predictive factor for possible NSAID hypersensitivity and that OPTs can be safely performed in these patients. However, as described in the European and American guidelines for aspirin provocation tests, caution and proper anaphylaxis surveillance is imperative when performing OPTs [21,23]. None of our patients had a history of delayed reactions or SCARs, which would be a contraindication of an OPT.

### NSAIDs involved

It has been debated whether patterns of consumption of NSAIDs are reflected in the prevalence of hypersensitivity reactions. A retrospective study by Dona et al. [24] involving 659 patients with NSAID hypersensitivity showed that the NSAIDs most likely to cause a hypersensitivity reaction changed over the course of three decades. Between 1980–1990 pyrazolones and ASA were the most frequent drugs involved in reactions. In the period from 1991–2000, ASA was the most frequent and reactions due to pyrazolones declined. Between 2001–2010 consumption of propionic acid derivatives such as ibuprofen increased dramatically and became the most frequent cause of NSAID hypersensitivity followed by ASA. Caimmi et al. [16] investigated 980 patients between 1998 and 2008 who were referred with NSAID hypersensitivity. The most common NSAIDs involved in the referrals were ASA (39%) and ibuprofen (15%). The patients in our cohort were examined from 2002 to 2011. Table 2 shows that ASA (40%), ibuprofen (32%) and diclofenac (13%) were the three most frequent incriminated causes of hypersensitivity reactions. No patients had a history of reactions to pyrazolones. A nationwide Danish study that investigated the pattern of use of NSAID in 4.6 million people between 1997 and 2005 [25], supports that there is a connection between consumption patterns of NSAID-classes and number of hypersensitivity reactions. The study investigated the amount of claimed prescriptions for NSAIDs (minus ASA). Ibuprofen and diclofenac were by far the two most frequently used NSAIDs in Denmark. Hardly any pyrazolones were used explaining the lack of referrals incriminating these drugs.

### Threshold dosages

When performing OPTs it is recommended that the cumulative dose should be at least the same level as the therapeutic dose [23]. The most recent EAACI/GA^2^LEN guideline on aspirin provocation tests recommend a cumulative dose of 500 mg when suspecting NSAIDs-exacerbated respiratory disease (NERD) and cumulative dose of 1000 mg when suspecting NSAIDs-exacerbated cutaneous disease (NECD) and multiple NSAIDs-induced urticaria/angioedema (NIUA) [21] Our OPT regime for ASA operates with a cumulative dose of 655 mg and our results support the use of high cumulative doses when performing OPTs (Table 1). The mean threshold dose for eliciting a hypersensitivity reaction upon OPT with ASA in 30 reactors was 483,5 mg. Recently Blanca-Lopez reported similar results in 57 positive ASA provocation tests with a median cumulative dose of 300 mg [17]. The cumulative dose of only 655 mg was a limitation in our study, however, our protocol was performed before the publication of the EAACI guidelines.

### Classification of OPT-positive patients

We succeeded in classifying 38/39 patients with proven NSAID hypersensitivity according to the EAACI/ ENDA classification (Table 4). The basis of this classification system is thorough knowledge of the clinical history and underlying diseases combined with the results of a positive OPT. The main challenge classifying the patients was collection of data concerning underlying diseases. Without data from our questionnaire, classification would not have been possible for all patients.

Still, classification was no simple task. Coexistence of cutaneous and airway symptoms in all three cross-reactive groups made classification a challenge. Four patients classified as NSAIDs-exacerbated respiratory disease (NERD) also had cutaneous symptoms upon OPT. However, as Samter acknowledged in his original work, NERD patients frequently also react with skin symptoms [26]. Similar findings were seen in the patients classified with NSAIDs-exacerbated cutaneous disease (NECD) and multiple NSAIDs-induced urticaria/angioedema (NIUA). In both of these two groups respiratory symptoms were often present concurrently with urticaria/angioedema (11 patients). Thus in total 15/39 (38.4%) in the three cross-reactive groups both had cutaneous and respiratory symptoms. The EAACI/ENDA-guideline and numerous other publications have previously reported overlaps in symptoms as being relatively rare [10,24,27,28]. Our results suggest that an overlap in symptoms may be more common than previously expected, and one can argue that the nomenclature of the cross-reactive immediate reactions i.e. NERD, NECD and NIUA can be misleading because symptoms from different organ systems can occur simultaneously.

Another challenging aspect classifying our patients was the coexistence of chronic urticaria and underlying respiratory disease. In our cohort 4 patients with chronic urticaria also suffered from nasal polyposis. Although this association is well described [24,29,30] it posed the following question: Should these patients be classified as NERD or NECD? We chose the latter based on the predominant urticarial reactions upon OPT.

Recently, Caimmi et al. addressed the problems with overlapping clinical manifestations and coexistence of underlying diseases when classifying 122 provocation positive patients according to the EAACI/ENDA-guidelines in a similar retrospective investigation [16]. Their solution was to create a new classification system with only three groups of immediate reactions opposed to four groups in the EAACI/ENDA-classification (Table 5). The NERD and NECD-groups were conjugated creating a common group for patients with underlying diseases. This puts less emphasis on the clinical manifestation of the hypersensitivity reaction. Joining the groups is possible because the putative mechanism of these groups is thought to be the same. Classifying our patients according to this system was easier although this system also had limitations. Hence we were unable to classify one patient in either of the classification systems. This patient was a child with no underlying diseases and a clinical history of urticaria in relation to her first ASA intake. OPT with ASA was positive and she was not tested with another NSAID. We could not establish if her reaction should be classified as multiple NSAID-induced urticaria/angioedema or as a single NSAID-induced reaction.

Research has shown that up to 30% of NSAID hypersensitivity reactions are selective [10,16,20,24]. In our study 9/39 (23.1%) could be classified as single NSAID induced urticaria/angioedema and anaphylaxis (SNIUAA). All patients had their hypersensitivity reaction to the culprit drug after an initial negative OPT with ASA. Diclofenac was the most common drug to cause reactions comprising 6 cases (66.7%). This prevalence is much higher than earlier reports [10,24]. Whether our findings reflect a higher degree of selective hypersensitivity to diclofenac than other NSAIDs is difficult to conclude based on the limited number of participants in this study. It might rather reflect regional differences in consumption of various NSAIDs worldwide. In Denmark, diclofenac is the second most prescribed NSAID [25]. The majority of literature about selective NSAID hypersensitivity consists of case reports so more studies are necessary before conclusions can be made. The only two cases of anaphylaxis in our cohort came after OPTs with diclofenac. This higher tendency of anaphylactic reactions among selective NSAID responders has previously been reported [8,31]. In our study no patients reacted to pyrazolone drugs. This is important because the notion that the putative mechanism behind single drug reactors is allergic primarily comes from observations on pyrazolones [11,12]. Using the definition for single drug reactors in the EAACI guidelines we found 9 patients with a positive reaction to a single NSAID (6 diclofenac, 2 ibuprofen, 1 naproxen) after a negative OPT with ASA. These reactions to non-pyrazolones would be suspected to be of non-allergic nature and the conclusion that single reactions to NSAIDs are of an allergic nature should therefore be taken very carefully in our patient cohort.

### Main limitations of this study

It is a retrospective study where data was collected from medical records over a long time-period of 10 years. We performed open provocation tests, which most guidelines do not recommend. For some patients, knowledge of underlying diseases such as asthma, nasal polyposis and CRS were based on a questionnaire, which is not optimal. Finally, in chronic urticaria it is well known that interpreting positive OPTs is difficult due to the risk of false positive results.

## Conclusions

This study underlines the importance of performing an OPT when establishing the diagnosis NSAID-hypersensitivity. The clinical history itself is not sufficient in an unselected patient cohort. However, the clinical history is important for numerous reasons. First of all, planning which OPTs should be performed is based on previous reactions to NSAIDs. Secondly, classifying NSAID-hypersensitive patients after a positive OPT is impossible without a detailed clinical history. It is a key feature that most hypersensitive patients suffer from underlying diseases such as asthma, CRS, nasal polyposis and chronic urticaria. The EACCI 2013 guideline recommend performing a challenge test with the culprit drug (if equivocal history) or to go directly to a challenge test with aspirin to exclude cross-reactivity. We performed a challenge test with aspirin directly to exclude cross-reactivity and found this approach partly helpful. However, the distinction between cross-reactors and single reactors was not easy when performing provocation tests with ASA and the culprit drug (if ASA was negative) only. Furthermore, overlaps in symptoms were common in the patients rendering classification within this system difficult.

## Abbreviations

NSAIDs: Non-steriodal anti-inflammatory drugs
OPT: Oral provocation test
SCARs: Severe adverse drug reactions
CRS: chronic rhinosinusitis
ASA: acetylsalicylic acid
NERD: NSAIDs-exacerbated respiratory disease
NECD: NSAIDs-exacerbated cutaneous disease
NIUA: Multiple NSAIDs-induced urticaria/angioedema
SNIUAA: Single NSAID-induced urticaria/angioedema and anaphylaxis

## References

[1] Gomes ER, Demoly P (2005). Epidemiology of hypersensitivity drug reactions. Curr Opin Allergy Clin Immunol.

[2] Jenneck C, Juergens U, Buecheler M, Novak N (2007). Pathogenesis, diagnosis, and treatment of aspirin intolerance. Ann Allergy Asthma Immunol.

[3] Gomes E, Cardoso MF, Praca F, Gomes L, Marino E, Demoly P (2004). Self-reported drug allergy in a general adult Portuguese population. Clin Exp Allergy.

[4] Erbagci Z (2004). Multiple NSAID intolerance in chronic idiopathic urticaria is correlated with delayed, pronounced and prolonged autoreactivity. J Dermatol.

[5] Kim JE, Kountakis SE (2007). The prevalence of Samter’s triad in patients undergoing functional endoscopic sinus surgery. Ear Nose Throat J.

[6] Canto MG, Andreu I, Fernandez J, Blanca M (2009). Selective immediate hypersensitivity reactions to NSAIDs. Curr Opin Allergy Clin Immunol.

[7] Asero R (2005). Oral aspirin challenges in patients with a history of intolerance to single non-steroidal anti-inflammatory drugs. Clin Exp Allergy.

[8] Quiralte J, Blanco C, Castillo R, Ortega N, Carrillo T (1997). Anaphylactoid reactions due to nonsteroidal antiinflammatory drugs: clinical and cross-reactivity studies. Ann Allergy Asthma Immunol.

[9] Stevenson DD, Sanchez-Borges M, Szczeklik A (2001). Classification of allergic and pseudoallergic reactions to drugs that inhibit cyclooxygenase enzymes. Ann Allergy Asthma Immunol.

[10] Quiralte J, Blanco C, Delgado J, Ortega N, Alcntara M, Castillo R (2007). Challenge-based clinical patterns of 223 Spanish patients with nonsteroidal anti-inflammatory-drug-induced-reactions. J Investig Allergol Clin Immunol.

[11] Kowalski ML, Makowska JS, Blanca M, Bavbek S, Bochenek G, Bousquet J (2011). Hypersensitivity to nonsteroidal anti-inflammatory drugs (NSAIDs) - classification, diagnosis and management: review of the EAACI/ENDA(#) and GA2LEN/HANNA*. Allergy.

[12] Kowalski ML, Asero R, Bavbek S, Blanca M, Blanca-Lopez N, Bochenek G (2013). Classification and practical approach to the diagnosis and management of hypersensitivity to nonsteroidal anti-inflammatory drugs. Allergy.

[13] Simons FE, Ardusso LR, Bilo MB, Dimov V, Ebisawa M, El-Gamal YM (2012). 2012 Update: world allergy organization guidelines for the assessment and management of anaphylaxis. Curr Opin Allergy Clin Immunol.

[14] Sampson HA (2003). Anaphylaxis and emergency treatment. Pediatrics.

[15] Tomassen P, Newson RB, Hoffmans R, Lotvall J, Cardell LO, Gunnbjornsdottir M (2011). Reliability of EP3OS symptom criteria and nasal endoscopy in the assessment of chronic rhinosinusitis–a GA(2) LEN study. Allergy.

[16] Caimmi S, Caimmi D, Bousquet PJ, Demoly P (2012). How can we better classify NSAID hypersensitivity reactions?–validation from a large database. Int Arch Allergy Immunol.

[17] Blanca-Lopez N, JT M, Dona I, Campo P, Rondon C, Seoane Reula ME (2013). Value of the clinical history in the diagnosis of urticaria/angioedema induced by NSAIDs with cross-intolerance. Clin Exp Allergy.

[18] Dursun AB, Woessner KA, Simon RA, Karasoy D, Stevenson DD (2008). Predicting outcomes of oral aspirin challenges in patients with asthma, nasal polyps, and chronic sinusitis. Ann Allergy Asthma Immunol.

[19] Schubert B, Grosse Perdekamp MT, Pfeuffer P, Raith P, Brocker EB, Trautmann A (2005). Nonsteroidal anti-inflammatory drug hypersensitivity: fable or reality?. Eur J Dermatol.

[20] Viola M, Rumi G, Valluzzi RL, Gaeta F, Caruso C, Romano A (2011). Assessing potential determinants of positive provocation tests in subjects with NSAID hypersensitivity. Clin Exp Allergy.

[21] Nizankowska-Mogilnicka E, Bochenek G, Mastalerz L, Swierczynska M, Picado C, Scadding G (2007). EAACI/GA2LEN guideline: aspirin provocation tests for diagnosis of aspirin hypersensitivity. Allergy.

[22] Hjortlund J, Mortz CG, Skov PS, Eller E, Poulsen JM, Borch JE (2012). One-week oral challenge with penicillin in diagnosis of penicillin allergy. Acta Derm Venereol.

[23] Macy E, Bernstein JA, Castells MC, Gawchik SM, Lee TH, Settipane RA (2007). Aspirin challenge and desensitization for aspirin-exacerbated respiratory disease: a practice paper. Ann Allergy Asthma Immunol.

[24] Dona I, Blanca-Lopez N, Cornejo-Garcia JA, Torres MJ, Laguna JJ, Fernandez J (2011). Characteristics of subjects experiencing hypersensitivity to non-steroidal anti-inflammatory drugs: patterns of response. Clin Exp Allergy.

[25] Fosbol EL, Gislason GH, Jacobsen S, Abildstrom SZ, Hansen ML, Schramm TK (2008). The pattern of use of non-steroidal anti-inflammatory drugs (NSAIDs) from 1997 to 2005: a nationwide study on 4.6 million people. Pharmacoepidemiol Drug Saf.

[26] Samter M, Beers RF (1967). Concerning the nature of intolerance to aspirin. J Allergy.

[27] Quiralte J, Blanco C, Castillo R, Delgado J, Carrillo T (1996). Intolerance to nonsteroidal antiinflammatory drugs: results of controlled drug challenges in 98 patients. J Allergy Clin Immunol.

[28] Ayuso P, Blanca-Lopez N, Dona I, Torres MJ, Gueant-Rodriguez RM, Canto G (2013). Advanced phenotyping in hypersensitivity drug reactions to NSAIDs. Clin Exp Allergy.

[29] Isik SR, Karakaya G, Celikel S, Demir AU, Kalyoncu AF (2009). Association between asthma, rhinitis and NSAID hypersensitivity in chronic urticaria patients and prevalence rates. Int Arch Allergy Immunol.

[30] Asero R, Madonini E (2006). Bronchial hyperresponsiveness is a common feature in patients with chronic urticaria. J Investig Allergol Clin Immunol.

[31] Chaudhry T, Hissaria P, Wiese M, Heddle R, Kette F, Smith WB (2012). Oral drug challenges in non-steroidal anti-inflammatory drug-induced urticaria, angioedema and anaphylaxis. Intern Med J.

